# Copula-based markov chain logistic regression modeling on binomial time series data

**DOI:** 10.1016/j.mex.2023.102509

**Published:** 2023-12-09

**Authors:** Pepi Novianti, Gunardi, Dedi Rosadi

**Affiliations:** a Department of Mathematics, Faculty of Mathematics and Natural Sciences, Universitas Gadjah Mada, Yogyakarta 55281, Indonesia; b Department of Statistics, Faculty of Mathematics and Natural Sciences, Bengkulu University, Bengkulu 38371, Indonesia

**Keywords:** Copula-Based Markov Chain Logistic Regression Model, Autoregressive, Asymptotic properties, Clayton, Conditional probability, Count time series, Frank, Gumbel, Maximum likelihood estimation

## Abstract

A first-order autoregressive time series model with binomial distributed random variables has been developed using the copula-based Markov chain model approach. By still utilizing conditional probability, covariate variables can also be included in the model and can be assumed as the independent variable. The time series dependent variable with a binomial distribution and continuous independent variables can be modelled using a copula-based Markov chain model with the probability of success expressed in the logit model. This study proposes a copula-based Markov chain logistic regression model with marginal binomial and joint distribution functions built through the copula function. Besides that, this study aims to estimate the parameters involved in the model. The parameters are the parameters of the logistic regression model as the relationship between the dependent and independent variables and the copula parameter as a time dependency. Using the bivariate copula functions are Clayton, Gumbel and Frank, the parameter estimation method is Maximum Likelihood Estimation (MLE). Simulations were carried out to see the efficiency of the parameter estimation and asymptotic results. Based on the simulation results, it was concluded that MLE provides an accurate estimate of the copula-based Markov chain logistic regression. In addition, the copula-based Markov chain logistic regression model can not only see the relationship between the independent and dependent variables but also provide an estimate of the time dependency of the dependent variable. The following are some of the proposed approach's highlights:

• This method proposes a binomial time series data model with covariate variables by combining the logistic regression model and the first-order Markov chain model.
• Parameter estimation in this model uses the Maximum Likelihood Estimation method.
• The model provides the possibility to see the relationship between variables and the time dependency.

## Graphical abstract

**Figure:**
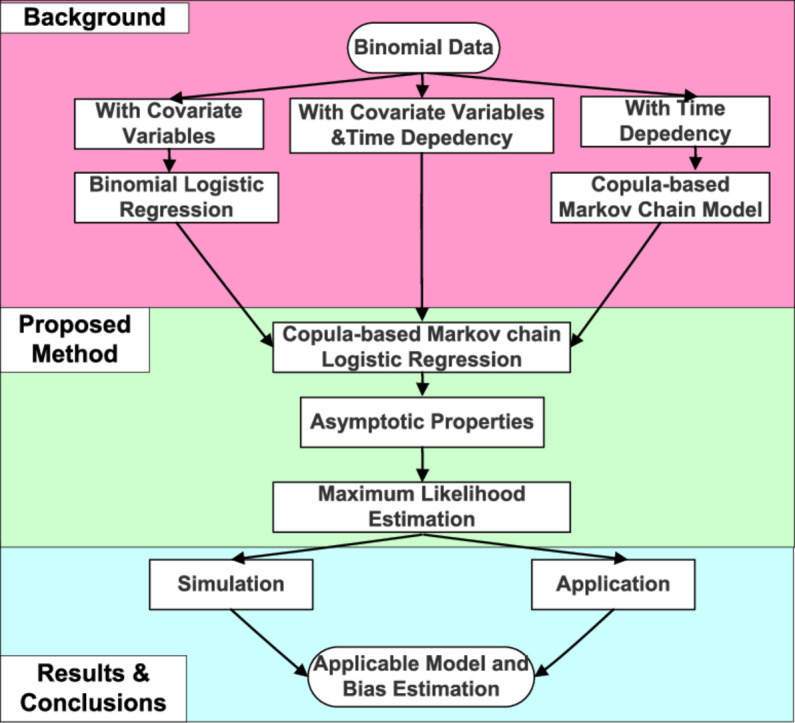

Specifications Table

**Table:** 

Subject area:	Mathematics and Statistics
More specific subject area:	Temporal Statistics, Count Time Series Data, Copula Modelling.
Name of your method:	Copula-Based Markov Chain Logistic Regression Model
Name and reference of original method:	Copula-based Markov zero-inflated count time series models with application
Resource availability:	Not applicable.

**Method details**

## Introduction

Kenzie (1986) discussed time series modelling with discrete random variables and used a first-order autoregressive structure to estimate the correlation between two adjacent observations, called INAR(1) [1]. Since discrete time series data have more complex features, the INAR(1) model is more complicated than the AR(1) model for continuous value time series models [1,2]. Karlis & Pedeli (2013) stated that INAR(1) has a limitation of such specifications in that it allows only for positive correlation between the two series [3]. INAR(1) model with binomial time series data is known as the binomial AR(1) model. Binomial time series model has been well applied to several real problems, such as in the fields of finance and industry [4,5]. Huang & Emura (2022) used a copula-based Markov in modelling binomial time series to overcome the complexity and limitation of Binomial AR(1) [5]. Apart from that, the copula method has several advantages, namely capturing dependencies between two time series, being used flexibly for discrete bivariate distributions, and allowing for a negative correlation [3]. Also, the marginals need not be in the same family and are easier to expand to an *n*-dimensional multivariate class employing the vine copula [6].

Binomial time series models in [1,^4^,5] are proposed on univariate binomial time series data with probability p. However, some data can be provided with covariate variables, which can be described as probability functions. Wu & Cui (2012) proposed a semiparametric method to obtain linear model parameter estimation which expresses the probability of success in the logit model [7], while Dunsmuir & He (2016) developed an approach that uses estimates based on one-dimensional marginal distribution through the generalized linear mixed model (GLMM) method [8]. Both approaches proposed parameter estimation in which time dependencies are expressed in latent processes and produce consistent and asymptotically normal regression parameter estimates.

Even though GLMM method can easily estimate parameters in a binomial time series model, time dependencies are neglected and are expressed in latent processes. Alqawba & Diawara (2020) stated that ignoring time dependencies can give inaccurate results and proposed a discrete time series model through a copula-based joint distribution of zero-inflated count time series observations [9]. The copula function is used in constructing the joint distribution function in Markov chains which was first introduced by Joe [10]. Many studies define copula-based predictive models as conditional expectations of the dependent variable given the independent variables [11], [12], [13], [14], [15]. Some important advantages of the copula-based Markov model are that it can avoid some of the tight distribution assumptions on marginal variables and can be extended to non-stationary processes through time-varying parameters within univariate margins of discrete distributions. In addition, the copula function can more easily handle *n*-dimensional joint distributions compared to multivariate joint distribution functions with certain distributions.

In this study, we modify copula-based Markov zero-inflated count time series model for binomial time series models with covariate variables modelled as the probability of success. The proposed model is called the copula-based Markov chain logistic regression model. Copula functions can capture time dependencies and the models used are the Clayton, Gumbel and Frank Copula. These three copulas are Archimedean family, which are frequently utilized in a variety of applications due to their closed forms, mathematical tractability and flexibility in capturing strong dependencies [16], [17], [18]. The probability of success of the marginal variable is determined from the inverse logit model based on the estimation results of the linear model parameters. We propose the Maximum Likelihood Estimation procedure for estimating model parameters, both logit parameter estimates and time dependency estimates. Firstly, we present computational parameter estimation and simulation to assess the performance of the proposed estimation method. Moreover, we apply the proposed method to model the relationships between climatic factors and influenza incidence in Singapore in 2012–2013.

## Model specifications and estimation procedures

### Copula

Copula is a function that combines the joint multivariate distribution function, $F(X_{1},\;.\;.\;.,X_{n})$, with its one-dimensional marginal distribution function, $F_{1}\left(X_{1}\right),\ldots$,$F_{n}\left(X_{n}\right)$, where the marginal distribution function is uniformly distributed over the range value [0,1]. A copula with only 2 joint distribution is called a bivariate copula or 2-dimensional copula. The basis of the copula is Sklar's theorem which states that the copula is a bivariate distribution function that has a uniform marginal distribution over the interval [0, 1]. Sklar's theorem explains the role that the copula plays in the relationship between its bivariate and univariate marginal distribution functions [19].

There are several methods for constructing bivariate Copula, including the Archimedean Copula which is widely used and is an important family in copula-based modelling. The copula model that will be used in this paper is the Archimedean Copula with one parameter, namely Clayton, Gumbel and Frank Copula.

### Clayton copula

Clayton Copula is an asymmetric Archimedean Copula which exhibits a greater dependency on the negative tail than on the positive tail. Clayton Copula has the following distribution function:

(1) $$C\left(u,\;v\right)=\max\left({\left(u^{-\theta }+v^{-\theta }-1\right)}^{-\frac{1}{\theta }}\theta ,\;0\right)$$

and the density is

(2) $$c\left(u,\;v\right)=\;\left(\theta \;+\;1\right){\left(uv\right)}^{-\left(\theta +1\right)}{\left(u^{-\theta }+v^{-\theta }-1\right)}^{-\frac{1+2\theta }{\theta }}$$

where θ∈[−1,∞)−0. Clayton copula has an upper tail dependency $\lambda_{U}=0$ and a lower tail dependency $\lambda_{L}\;=\;2^{\left(-\frac{1}{\theta }\right)}$. The conditional distribution of Clayton Copula is given by:

(3) $$C\left(u|v;\alpha \right)=v^{-\alpha -1}{\left(u^{-\alpha }+v^{-\alpha }-1\right)}^{-\frac{1}{\alpha }-1}$$

### Gumbel Copula

In contrast to Clayton Copula, Gumbel Copula has a lower tail dependency $\lambda_{L}=0$ and an upper tail dependency $\lambda_{U}=2^{\left(-\frac{1}{\theta }\right)}$, hence this copula shows greater dependency on the positive tail. Gumbel Copula has the distribution function is

(4) $$C\left(u,v\right)=e^{-{\left[{\left(-\ln u\;\right)}^{\theta }+{\left(-\ln v\right)}^{\theta }\right]}^{\frac{1}{\theta }}}$$

and the density function is

(5) $$c\left(u,v\right)=\frac{1}{u}\frac{1}{v}{\left(-\ln u\;\right)}^{\theta -1}{\left(-\ln v\;\right)}^{\theta -1}w^{\frac{1}{\theta }-2}C\left(u,v\right)\left(w^{\frac{1}{\theta }}+\left(\theta -1\right)\right)$$

with $w=-[{\left(-\ln u\;\right)}^{\theta }+{\left(-\ln v\right)}^{\theta }]$ and θ∈[−1,∞). The conditional distribution of Gumbel Copula is given by:

(6) $$C\left(u|v;\theta \right)=\frac{1}{v}e^{-{\left[{\left(-\ln u\;\right)}^{\theta }+{\left(-\ln v\right)}^{\theta }\right]}^{\frac{1}{\theta }}{\left[1+{\left(\frac{-\ln u}{-\ln v}\right)}^{\theta }\right]}^{\frac{1}{\theta }-1}}$$

### Frank copula

Frank copula exhibits a parallel correlation structure and is the only Archimedean copula that has a symmetrical shape. Frank copula has positive and negative tail dependencies with a value of zero. For Frank Copula, the distribution function is:

(7) $$C\left(u,v\right)=-\frac{1}{\theta }\ln\left(1+\frac{\left(e^{-\theta u}-1\right)\left(e^{-\theta v}-1\right)}{e^{-\theta }-1}\right)$$

and the probability distribution function is

(8) $$c\left(u,v\right)=\frac{\theta \left(e^{-\theta }-1\right)e^{-\theta \left(u+v\right)}}{e^{-\theta }-1}$$

The conditional distribution of Frank Copula is given by:

(9) $$C\left(u|v;\theta \right)=e^{-\theta v{\left[\left(e^{-\theta }-1\right){\left(e^{\theta u}-1\right)}^{-1}+\left(e^{\theta v}-1\right)\right]}^{-1}}$$

### Binomial logistic regression

Suppose the response vector $Y={\left(Y_{1},\ldots ,Y_{T}\right)}^{T}$ is assumed to have a binomial distribution. For binomial data, the observed response for the i*^th^* observation, i=1,2,...,T, is the proportion denoted by $\frac{y_{i}}{n_{i}}$. The response corresponding to the i*^th^* observation is the binomial distribution $B(n_{i},\pi_{i})$, where $\pi_{i}$ is the probability of success or response probability and $n_{i}$ is the total of observations. Therefore, the expected value for the response variable is $E\left(Y_{i}\right)=n_{i}\pi_{i}$ and $Var\left(Y_{i}\right)=n_{i}\pi_{i}\left(1-\pi_{i}\right)$.

In logistic regression, the model is explored in the probability of success in the i*^th^* observation, $\pi_{i}=E\left(\;\frac{Y_{i}}{n_{i}}\right)$ which value in the range (0,1). To ensure that the probability is between 0 and 1, a logistic transformation is used to model the linear equation. Logistic or logit transformation is a transformation of the probability of success p which is written as [16].

(10) $$logit\left(\pi \right)=\log\left(\frac{\pi }{1\;-\;\pi }\right)=\beta_{0}+\beta_{1}x_{1}+\cdot \cdot \cdot +\beta_{d}x_{d}$$

and can be realigned into the success probability equation shown below:

(11) $$\pi \;=\frac{\exp\left(\beta_{0}+\beta_{1}x_{1}+\cdot \cdot \cdot +\beta_{d}x_{d}\right)}{1+\exp\left(\beta_{0}+\beta_{1}x_{1}+\cdot \cdot \cdot +\beta_{d}x_{d}\right)}=\frac{\exp\left(X\beta \right)}{1+\exp\left(X\beta \right)}$$

### Copul-based Markov Chain autoregressive logistic regression model

The general form of the first-order Markov model with the transition probability is defined as

(12) $$Y_{t}=\;g\left(\varepsilon_{t};Y_{t-1}\right)$$

where $\varepsilon_{t}$ is the stochastic continuous latent process *i.i.d* and g(·) is assumed to be an increasing function at $\varepsilon_{t}$ for t=1,…,T. Therefore, the observed values of $Y_{t}$ depend on the past only through $Y_{t-1}$. If the $Y_{t}$ process is continuous, then there is a simple stochastic representation for the Markov model. However, for discrete processes, the stochastic representation of the model becomes more complicated.

Let $Y_{t}$ be a discrete time series following a first-order Markov chain. By utilizing the probability chain rule and the Markov property, the multivariate joint probability distribution of $Y_{1},\ldots ,Y_{T}$ is given as

(13) $$\mathrm{Pr}\left(Y_{1}=y_{1},\ldots ,Y_{T}=y_{T}\right)=\mathrm{Pr}\left(Y_{1}=y_{1}\right)\prod_{t=2}^{T}\mathrm{Pr}\left(Y_{t}=y_{t}|Y_{t-1}=y_{t-1}\right)$$

The transition probability depends on the joint probability function of $\left(Y_{t},Y_{t-1}\right)$ and can be determined using the copula function. Thus, the joint cumulative function with margin $Y_{t}$ and $Y_{t-1}$ is expressed as

(14) $$F\left(Y_{t},Y_{t-1}\right)=C\left(F\left(y_{t}|X_{t};\beta \right),F\;\left(y_{t-1}|X_{t-1};\beta \right)\right);\alpha )$$

where C(·,·;α) is a bivariate copula function with parameter α. Covariate $X_{t}=\left(1,X_{1t},\ldots ,\;X_{dt}\right)$, for t=1,…,T, is the covariate corresponding to the probability parameter $\pi_{t}$. The parameter vector $\beta =(\beta_{0},\beta_{1},\ldots ,\beta_{d})$ is the unknown marginal regression coefficient. Therefore, the transition probability is given as

(15) $$\mathrm{Pr}\left(Y_{t}=y_{t}|Y_{t-1}=y_{t-1}\right)=\frac{\mathrm{Pr}\left(Y_{t}=y_{t},Y_{t-1}=y_{t-1}\right)}{\mathrm{Pr}\left(Y_{t-1}=y_{t-1}\right)}$$

where $\mathrm{Pr}\left(Y_{t-1}=y_{t-1}\right)=f_{t-1}\left(y_{t-1}|X_{t-1};\;\beta \right)$ and $Pr\left(Y_{t}=y_{t},Y_{t-1}=y_{t-1}\right)=C\left(u_{t},v_{t};\alpha \right)-C\left(u_{t},v_{t}^{-};\alpha \right)-C\left(u_{t}^{-},v_{t};\alpha \right)+C\left(u_{t}^{-},v_{t}^{-};\alpha \right)$ with $u_{t}=F\left(y_{t}|X_{t};\beta \right)$, $v_{t}=F\left(y_{t-1}|X_{t-1};\beta \right)$, $u_{t}^{-}=F\left(y_{t}-1|X_{t};\beta \right)$ and $v_{t}^{-}=F\left(y_{t-1}-1|X_{t-1};\beta \right)$ [16], [17], [18].

Let $Y={\left(Y_{1},\ldots ,Y_{T}\right)}^{T}$ is a vector of the binomial time series data observed at T time points, and $X={\left(X_{1},\ldots ,X_{T}\right)}^{T}$ is the covariate matrix T×(d+1), where $X_{t}=\left(1,\;X_{1t},\ldots ,\;X_{dt}\right)$ for t=1,…,T and d is the number of covariate variables. The binomial logistic regression model for discrete time series data is defined in Eq. (11), where $\beta ={\left(\beta_{0},\beta_{1},\ldots ,\beta_{d}\right)}^{T}$ is the parameter coefficient vector of the logistic regression model to be estimated. From this regression model, ${\hat{\pi }}_{t}=\frac{\exp(X_{t}\beta )}{1+\exp(X_{t}\beta )}\;$is the success probability for the marginal variable $Y_{t}$ which has a binomial distribution. Therefore, the probability function and distribution of the variable $Y_{t}\;$is

(16) $$f_{t}\left(y_{t}|X_{t};\beta \right)=\left(\begin{array}{c} n_{t}\\ y_{t} \end{array}\right){\hat{\pi }}_{t}^{y_{t}}{\left(1-{\hat{\pi }}_{t}\right)}^{n_{t}-y_{t}}$$

and

(17) $$F_{t}\left(y_{t}|X_{t};\beta \right)=\sum_{k=0}^{y_{t}}f_{t}\left(k_{t}|X_{t};\beta \right)$$

It is also assumed in this section that the discrete time series $\left\{Y_{t}|X_{t};\;\beta ,t=1,2,\ldots ,n\right\}$ are a first order Markov process with discrete state space, that is, $Y_{t}|Y_{t-1},X_{t};\;\beta ,t=1,2,\ldots ,n.$ Under this assumption, the time series probabilistic property is determined by the joint distribution of $Y_{t}$ and $Y_{t-1}$ and is denoted as $F(Y_{t},Y_{t-1}|X_{t},\;\beta )$. This joint distribution can be determined based on the bivariate copula model, so that for $\forall (y_{t},y_{t-1})\in R$ has a joint cumulative distribution as in Eq. (14), where C(·,·;α) is the copula function that does not vary with the covariate, and α is the unknown copula parameter.

For continuous time series, by Sklar's theorem $F(Y_{t},Y_{t-1}|X_{t},\;\beta )$ can be represented by the marginal conditional distribution function $F(Y_{t}|X_{t},\;\beta )$ of $Y_{t}$ and the unique copula function C(·,·;α). However, when the marginal distribution function F(·) is discrete data, the joint distribution F(·,·) is uniquely defined only at certain intervals. With logistic regression model $Y_{t}$ on covariate $X_{t}$ which is continuous, it is possible to expand the value of $F(Y_{t}|X_{t},\;\beta )$ from a number of discrete points into intervals. Thus, it can be ensured that the function of the copula can be determined uniquely by the population in all regions from the possible values of $F(Y_{t}|X_{t},\;\beta )$. This is summarized in the following statement:

Proposition 1 Suppose $Y={\left(Y_{1},\ldots ,Y_{n}\right)}^{T}$ is a binomial time series data vector and $Y_{t}$ is characterized by binomial logistic regression with a marginal distribution of $F(y_{t}|X_{t};\beta )$ and satisfies the first-order markov process $\text{Pr}\left(Y_{t}\leq y_{t}|Y_{t-1},...,Y_{1},X\right)=Pr\left(Y_{t}\leq y_{t}|Y_{t-1},X\right)$. Suppose that there is also a bivariate copula C(·,·;α) such that for $\forall (y_{t},y_{t-1})\in R$ has a joint cumulative distribution

(18) $$F\left(y_{t},y_{t-1}|x\right)=\;C\left(F\left(y_{t}|X_{t};\beta \right),\;F\left(y_{t-1}|X_{t-11};\beta \right);\;\alpha \right)$$

where C(·,·;α) is the copula function that does not vary with the covariate, and α is the unknown copula parameter. If at least one of the covariates x is continuous with nonzero coefficients in the marginal distribution $F(\cdot |X_{t};\beta )$, then the copula function C(·,·;α) can be uniquely determined on the interval [0,1].

Proof of Proposition 1 It is assumed that Y is binomial logistic regression binomial time series data vector with marginal distribution $F(y_{t}|X_{t};\beta )$. Suppose one of the covariates $X_{t}$ in X is continuous with non-zero coefficients, then $\left(Y_{t}|X_{t};\beta \right)∼Bin\left(n_{t},\pi_{t}\right)$ and $\left(Y_{t-1}|X_{t-1};\beta \right)∼Bin\left(n_{t-1},\pi_{t-1}\right)$. Thus obtained

(19) $$log\left(\frac{\pi_{t}}{1+\pi_{t}}\right)=\;X_{t}\beta$$

(20) $$log\left(\frac{\pi_{t-1}}{1+\pi_{t-1}}\right)=\;X_{t-1}\beta$$

$Y_{t}$ is a 1^st^ order markov process, so $Y_{t}$ and $Y_{t-1}$ are not mutually exclusive. Let *C* be a copula, then

(21) $$F\left(Y_{t},\;Y_{t-1}|x\right)=\;C\left(F\left(y_{t}|X_{t};\beta \right),F\left(y_{t-1}|X_{t-11};\beta \right);\;\alpha \right)$$

apply. Therefore, for $\forall C_{1},\;C_{2}\in C,$ and ∀u,v∈[0,1]×[0,1], using triangle inequality we get

$$|C_{1}\left(u,\;v\right)\;-\;C_{2}\left(u,\;v\right)\left|\;\leq \right|C_{1}\left(u,\;v\right)\;-\;C_{1}(F\left(y_{t}|x\right),\;F\left(y_{t-1}|x\right)\left|+\;\right|C_{2}\left(u,\;v\right)\;-\;C_{2}\left(F\left(y_{t}|x\right),\;F\left(y_{t-1}|x\right)\right|$$

Since X is a continuous random variable, $F(y_{t}|x)$ and $F(y_{t-1}|x)$ can take on all values in (0,1). Therefore, for ∀u,v∈(0,1), there are $X_{t}$, $X_{t-1}$*,* $y_{t}$, $y_{t-1}$, where $y_{t}$ depend on $X_{t}$ and $y_{t-1}$ depend on $X_{t-1}$, so $F(y_{t}|X_{t};\beta )=u$ and $F(y_{t-1}|x)=v$, and consequently

(22) $$\begin{array}{c} |C_{1}\left(u,\;v\right)\;-\;C_{2}\left(u,\;v\right)\left|\;\leq \right|C_{1}\left(u,\;v\right)\;-\;C_{1}(F\left(y_{t}|x\right),\;F\left(y_{t-1}|x\right)\left|+\;\right|C_{2}\left(u,\;v\right)\;-\;C_{2}(F\left(y_{t}|x\right),\;F\left(y_{t-1}|x\right)\\ \left|C_{1}\left(u,\;v\right)-\;C_{2}\left(u,\;v\right)\right|\leq 0|\\ C_{1}\left(u,\;v\right)-\;C_{2}\left(u,\;v\right)=0\\ C_{1}\left(u,\;v\right)=C_{2}\left(u,\;v\right) \end{array}$$

Therefore, the function of copula C(·,·;α) can be determined uniquely at the interval [0, 1]. ■

### Estimation parameter

The parameter vector of the copula-based Markov Chain Logistic Regression model for the binomial data is estimated by the maximum likelihood estimation (MLE) method. This method is easy to apply when the selected copula family has a closed-form and provides advantages in model selection through the log-likelihood function. By using the transition probability in Eq. (15) and expressing the likelihood function in logarithms, the following log-likelihood function is obtained:

(23) $$\begin{array}{c} L\left(\beta ,\alpha \right)=\log\left[\mathrm{Pr}\left(Y_{1}=y_{1}\right)\prod_{t=2}^{n}\mathrm{Pr}\left(Y_{t}=y_{t}|Y_{t-1}=y_{t-1}\right)\right]\\ \log\left[f\left(y_{1}|X_{1};\;\beta \right)\prod_{t=2}^{n}\frac{\mathrm{Pr}\left(Y_{t}=y_{t},Y_{t-1}=y_{t-1}\right)}{f_{t-1}\left(y_{t-1}|X_{t-1};\;\beta \right)}\right]\\ \sum_{t=2}^{n}\log\left[C\left(u_{t},v_{t};\alpha \right)-C\left(u_{t},v_{t}^{-};\alpha \right)-C\left(u_{t}^{-},v_{t};\alpha \right)+C\left(u_{t}^{-},v_{t}^{-};\alpha \right)\right]-\sum_{t=2}^{n-1}\log\left[f_{t-1}\left(y_{t-1}|X_{t-1};\;\beta \right)\right] \end{array}$$

Based on the approach in [9], then the estimation of the probability parameters β and α in the AR(1) Binomial model are obtaioned simultaneously by maximizing the log-likelihood value in Eq. (23) or

(24) $$\left(\hat{\beta },\hat{\alpha }\;\right)=\underset{\beta ,\alpha }{\text{argmax}}L\left(\beta ,\alpha \right)$$

where α is the copula parameter and β is the logistic regression marginal parameter.

When estimating parameters, the log-likelihood function L(β,α) is optimized, resulting in a Hessian Matrix. The observed Fisher information matrix of MLE $\left(\hat{\beta },\hat{\alpha }\;\right)$ which can be used to calculate the standard error is produced by the Hessian matrix. To obtain the parameter estimation$\;(\hat{\beta },\hat{\alpha }\;)$ using MLE, the scoring function is required which is stated in the following lemma:

Lemma 1 Suppose the likelihood function of copula-based Markov Chain logistic regression model for binomial data is given by l(β,α), where $\hat{\theta }=\left(\hat{\beta },\hat{\alpha }\right)$ is the maximum likelihood estimator, then the score function of the copula-based Markov Chain logistic regression model is

(24) $$S\left(\theta \right)=\left[\frac{\partial l\left(\beta ,\alpha \right)}{\partial \beta_{0}},\frac{\partial l\left(\beta ,\alpha \right)}{\partial \beta_{1}},\cdots ,\frac{\partial l\left(\beta ,\alpha \right)}{\partial \beta_{d}},\frac{\partial l\left(\beta ,\alpha \right)}{\partial \alpha }\right]$$

Where

(25) $$\frac{\partial l\left(\beta ,\alpha \right)}{\partial \beta_{j}}=\sum_{t=2}^{n}\frac{\Delta {∁}_{\beta_{j}t}^{\prime }}{\Delta {∁}_{t}}-\left(T-2\right)x_{j}$$

(26) $$\frac{\partial l\left(\beta ,\alpha \right)}{\partial l\left(\alpha \right)}=\sum_{t=2}^{T}\frac{\Delta C_{t,\alpha }^{\prime }}{\Delta C_{t}}$$

With

$$\begin{array}{c} \Delta C_{t}=C\left(u_{t},v_{t};\alpha \right)-C\left(u_{t},v_{t}^{-};\alpha \right)-C\left(u_{t}^{-},v_{t};\alpha \right)+C\left(u_{t}^{-},v_{t}^{-};\alpha \right),\\ \Delta C_{t,\beta }^{\prime }=x_{j}[u_{t}\left[C\left(v_{t}|u_{t};\alpha \right)-C\left(v_{t}-1|u_{t};\alpha \right)\right]+v_{t}\left[C\left(u_{t}|v_{t};\alpha \right)-C\left(u_{t}-1|v_{t};\alpha \right)\right]-\left(v_{t}-1\right)\left[C\left(u_{t}|v_{t}-1;\alpha \right)-C\left(u_{t}-1|v_{t}-1;\alpha \right)\right]\\ \;\;-\left(u_{t}-1\right)\left[C\left(v_{t}|u_{t}-1;\alpha \right)-C\left(v_{t}-1|u_{t}-1;\alpha \right)\right]] \end{array}$$

$\Delta C_{t,\beta }^{\prime }$ and $\Delta C_{t,\alpha }^{\prime }$ are partial derivative of $\Delta C_{t}$ with respect to the marginal parameter β and α respectively. C(·,·;α) is bivariate copula function, hence conditional copula function C(·|·;α) and partial derivative $\Delta C_{t,\alpha }^{\prime }$ depend on used copula function.

Proof of Lemma 1: The log-likelihood function of copula-based Markov Chain logistic regression model for binomial data is

$$L\left(\beta ,\alpha \right)=\sum_{t=2}^{n}\log\left[C\left(u_{t},v_{t};\alpha \right)-C\left(u_{t},v_{t}^{-};\alpha \right)-C\left(u_{t}^{-},v_{t};\alpha \right)+C\left(u_{t}^{-},v_{t}^{-};\alpha \right)\right]-\sum_{t=2}^{n-1}\log\left[f_{t-1}\left(y_{t-1}|X_{t-1};\;\beta \right)\right]$$

Firstly, we will derive the log-likelihood function for the marginal parameter $\beta_{j}$. Let $\Delta C_{t}=C\left(u_{t},v_{t};\alpha \right)-C\left(u_{t},v_{t}-1;\alpha \right)-C\left(u_{t}-1,v_{t};\alpha \right)+C\left(u_{t}-1,v_{t}-1;\alpha \right)$, its partial derivative with respect to the marginal parameter β is given by:

(27) $$\begin{array}{ccc} \frac{\partial \Delta C}{\partial \beta_{j}} & = & \frac{\partial \left[C\left(u_{t},v_{t};\alpha \right)-C\left(u_{t},v_{t}-1;\alpha \right)C\left(u_{t}-1,v_{t};\alpha \right)+C\left(u_{t}-1,v_{t}-1;\alpha \right)\right]}{\partial \beta_{j}}\\ \frac{\partial \Delta C}{\partial \beta_{j}} & = & \frac{\partial C\left(u_{t},v_{t};\alpha \right)}{\partial \beta_{j}}-\frac{\partial \left(u_{t}|v_{t}^{-};\alpha \right)}{\partial \beta_{j}}-\frac{\partial C\left(u_{t}^{-}|v_{t};\alpha \right)}{\partial \beta_{j}}+\frac{\partial C\left(v_{t}^{-}|u_{t}^{-};\alpha \right)}{\partial \beta_{j}}\\ \frac{\partial \Delta C}{\partial \beta_{j}} & = & x_{j}[u_{t}\left(C\left(v_{t}|u_{t};\alpha \right)-C\left(v_{t}^{-}|u_{t};\alpha \right)\right)+v_{t}\left(C\left(u_{t}|v_{t};\alpha \right)-C\left(u_{t}^{-}|v_{t};\alpha \right)\right)-u_{t}^{-}(C\left(v_{t}^{-}|u_{t}^{-};\alpha \right)-\left(C\left(v_{t}^{-}|u_{t}^{-};\alpha \right)\right)-v_{t}^{-}(C\left(u_{t}|v_{t}^{-};\alpha \right)\\ & & +C\left(u_{t}^{-}|v_{t}^{-};\alpha \right))] \end{array}$$

C(·|·;α) known as the conditional copula function is the partial derivative of bivariate copula function, C(·,·;α), with respect to the marginal distribution function.

For $f_{t}$, the marginal pdf for binomial distribution given in Eq (16), its partial derivative with respect to the marginal parameter β is

(28) $$\begin{array}{c} f_{t,\beta }^{\prime }=\frac{\partial f_{t}}{\partial \beta }=\frac{\partial f_{t}}{\partial \pi }\frac{\partial \pi }{\partial \beta }\\ f_{t,\beta }^{\prime }=\left(\begin{array}{c} n\\ y \end{array}\right)\frac{\pi^{y}{\left(1-\pi \right)}^{n-y}}{\pi \left(1-\pi \right)}\left(x_{j}\left(\frac{e^{X\beta }}{1+e^{X\beta }}\right)-x_{j}{\left(\frac{e^{X\beta }}{1+e^{X\beta }}\right)}^{2}\right)\\ f_{t,\beta }^{\prime }=\left(\begin{array}{c} n\\ y \end{array}\right)\frac{\pi^{y}{\left(1-\pi \right)}^{n-y}}{\pi \left(1-\pi \right)}\left(x_{j}\pi -x_{j}\pi^{2}\right)\\ f_{t,\beta }^{\prime }=\left(\begin{array}{c} n\\ y \end{array}\right)x_{j}\pi^{y}{\left(1-\pi \right)}^{n-y}\\ f_{t,\beta }^{\prime }=\left(\begin{array}{c} n\\ y \end{array}\right)\frac{\pi^{y}{\left(1-\pi \right)}^{n-y}}{\pi \left(1-\pi \right)}\left(x_{j}\left(\frac{e^{X\beta }}{1+e^{X\beta }}\right)-x_{j}{\left(\frac{e^{X\beta }}{1+e^{X\beta }}\right)}^{2}\right)\\ f_{t,\beta }^{\prime }=\left(\begin{array}{c} n\\ y \end{array}\right)\frac{\pi^{y}{\left(1-\pi \right)}^{n-y}}{\pi \left(1-\pi \right)}\left(x_{j}\pi -x_{j}\pi^{2}\right)\\ f_{t,\beta }^{\prime }=\left(\begin{array}{c} n\\ y \end{array}\right)x_{j}\pi^{y}{\left(1-\pi \right)}^{n-y} \end{array}$$

Therefore, partial derivative of our log-likelihood function with respect to the marginal parameter β is

(29) $$\begin{array}{c} \frac{\partial L\left(\beta ,\alpha \right)}{\partial \beta }=\frac{\partial \sum_{t=2}^{n}\log\Delta {∁}_{t}}{\partial \beta }-\frac{\partial \sum_{t=2}^{n-1}\log\left[f_{t-1}\right]}{\partial \beta }\\ \frac{\partial L\left(\beta ,\alpha \right)}{\partial \beta }=\sum_{t=2}^{n}\frac{\partial \log\Delta {∁}_{t}}{\partial \beta }-\sum_{t=2}^{n-1}\frac{\partial \log\left[f_{t-1}\right]}{\partial \beta }\\ \frac{\partial L\left(\beta ,\alpha \right)}{\partial \beta }=\sum_{t=2}^{n}\frac{\partial \Delta {∁}_{t}/\partial \beta }{\Delta {∁}_{t}}-\sum_{t=2}^{n-1}\frac{\partial \left[f_{t-1}\right]/\partial \beta }{\left[f_{t-1}\right]}\\ \frac{\partial L\left(\beta ,\alpha \right)}{\partial \beta }=\sum_{t=2}^{n}\frac{\Delta {∁}_{\beta_{j}t}^{\prime }}{\Delta {∁}_{t}}-\sum_{t=1}^{T-2}\frac{\left(\begin{array}{c} n_{t}\\ y_{t} \end{array}\right)x_{j}\pi_{t}^{y_{t}}{\left(1-\pi_{t}\right)}^{n_{t}-y_{t}}}{\left(\begin{array}{c} n_{t}\\ y_{t} \end{array}\right)\pi_{t}^{y_{t}}{\left(1-\pi_{t}\right)}^{n_{t}-y_{t}}}\\ \frac{\partial L\left(\beta ,\alpha \right)}{\partial \beta }=\sum_{t=2}^{n}\frac{\Delta {∁}_{\beta_{j}t}^{\prime }}{\Delta {∁}_{t}}-\left(T-2\right)x_{j} \end{array}$$

The next is to derive a partial derivative of the log-likelihood function with respect to the copula parameter α. Since the probability mass function $f_{t}$ does not contain a parameter component α, then $\frac{\partial f_{t}}{\partial \alpha }=\;0$ and $\frac{\partial L(\beta ,\alpha )}{\partial \alpha }$ is only determined from $\frac{\partial \Delta C}{\partial \alpha }$. The partial derivative of $C(v_{t},u_{t};\alpha )$ with respect to parameter α depends on the copula model used. Therefore, in general, the partial derivative of the log-likelihood function of copula-based Markov chain Logistic regression model for binomial data to the copula parameter α is obtained as follows

(30) $$\begin{array}{ccc} \frac{\partial L\left(\beta ,\alpha \right)}{\partial \alpha } & = & \frac{\partial \sum_{t=2}^{T}\log\Delta C_{t}}{\partial \Delta \alpha }=\sum_{t=2}^{T}\frac{\partial \log\Delta C_{t}}{\partial \Delta \alpha }\\ & = & \sum_{t=2}^{T}\frac{\partial \Delta C_{t}/\partial \alpha }{\Delta C_{t}}=\sum_{t=2}^{T}\frac{\Delta C_{t,\alpha }^{\prime }}{\Delta C_{t}} \end{array}$$

where $\Delta C_{t,\alpha }^{\prime }=\frac{\partial C(u_{t},v_{t};\alpha )}{\partial \alpha }-\frac{\partial (u_{t}|v_{t}^{-};\alpha )}{\partial \alpha }-\frac{\partial C(u_{t}^{-}|v_{t};\alpha )}{\partial \alpha }+\frac{\partial C(v_{t}^{-}|u_{t}^{-};\alpha )}{\partial \alpha }$ and $\frac{\partial C}{\partial \alpha }$ are determined by the copula model used. ■

### Asymptotic Properties

Billingsley (1961) in [9,20] stated that there are several conditions that fulfill the consistency and asymptotic normality properties of MLE estimation to be applied in copula-based Markov chain models, namely:

1. The maximum likelihood estimate (β,α) are obtained from solving optimization of the score function.
2. All states of the Markov chain communicate with each other (meaning that there are no transient states).
3. The set of y for which Pr(y|x;(β,α)) is positive does not depend on (β,α).
4. l(β,α) is continuous and two times continuously differentiable.
5. For θ=(β,α)∈Θ, there exists a neighborhood $N_{\theta }$ of θ such that for all i,j,
  (31) $$E_{\theta }\left[\underset{\theta^{\prime }\in N_{\theta }}{\sup}\left|p_{ij}\left(\theta^{\prime },Y_{t},Y_{t-1}\right)\right|\right]<\infty$$
  where $E_{\theta }$ means expectation assuming that the true parameter value is θ and $Y_{1}$ start with a stationary distribution.
6. $F(Y_{t}|X_{t},\;\beta )$ is absolutely continuous with respect to $f_{t}\left(y_{t}|X_{t};\beta \right)$.

With the given regularity conditions, asymptotic results are the following:

Theorem 1 Suppose that regularity conditions hold with the log-likelihood function

(32) $$L\left(\beta ,\alpha \right)=\sum_{t=2}^{n}\log C\left(u_{t},v_{t};\alpha \right)-C\left(u_{t},v_{t}^{-};\alpha \right)-C\left(u_{t}^{-},v_{t};\alpha \right)+C\left(u_{t}^{-},v_{t}^{-};\alpha \right)]-\sum_{t=2}^{n-1}\log\left[f_{t-1}\left(y_{t-1}|X_{t-1};\;\beta \right)\right]$$

And there exists a root (β,α) of $\frac{\partial l(\beta ,\alpha )}{\partial (\beta ,\alpha )}=0$, such that we have

(i) ${\hat{\theta }}_{P}\to \theta_{0}$ (consistent estimator)
(ii) $n^{1/2}\left({\hat{\theta }}_{P}-\theta_{0}\right)\overset{d}{\to }N_{P}\left(0,\Sigma^{-1}\left(\theta_{0}\right)\right)$ (asymptotically normal)

### Computation and simulation

This section discusses computation and simulation for parameter estimation of the Copula-Based Markov Chain Autoregressive Logistic Regression model. Computations are set up to calculate the log-likelihood function and determine the parameter values that optimize the function. Simulations are carried out on the generated data to see the performance of parameter estimation. Therefore, it is necessary to develop an algorithm for parameter estimation and data generation. The computational procedure to estimate parameters is explained as follows:

1. Input data in the form of the dependent variable Y, total sample N and the independent variable X.
2. Define the success probability function $\pi_{t}$ of dependent variable$\;Y_{t}$ based on independent variable $X_{t}$ using Eq. (11).
3. Define the probability function $f(y_{t};n_{t},{\hat{\pi }}_{t})\;$of the marginal variable using Eq. (16) with the success probability in step (2)
4. Define the cumulative distribution function $F(y_{t};n_{t},{\hat{\pi }}_{t})$ of the binomial marginal variables using Eq. (17).
5. Arrange the marginal distribution function $u_{t}$, $v_{t}$, $\left(u_{t}-1\right)$ and $\left(v_{t}-1\right)$.
6. Define the joint cumulative function using Eq. (14).
7. Define the log-likelihood function based on Eq. (23).
8. Set the initial value of the logistic regression parameters $\beta_{0}=\left(0,0,\ldots ,0\right)$ and the copula parameter$\;\alpha_{0}$.
9. Get the best parameter estimations $\hat{\beta }$ and $\hat{\alpha }$ that produce the largest copula Loglikelihood value.

The procedure for generating data refers to [21] which generates data from the Markov process with bivariate copula and Poisson regression model. The steps are explained as follow:

1. Generate the data sequences ${\left\{v_{t}\right\}}_{t=1}^{T}∼Unif\;\left(0,\;1\right)$, ${\left\{X_{1t}\right\}}_{t=1}^{T}∼Unif\left(70,\;100\right)$, ${\left\{X_{2t}\right\}}_{t=1}^{T}∼Unif\left(0,\;1\right)$ and ${\left\{n_{t}\right\}}_{t=1}^{T}∼Unif\left(200,\;500\right)$.
2. Determine $U_{1}=v_{1}$ and $U_{t}=C_{2|1}^{-1}\left(v_{t}\;|U=U_{t-1};\alpha \right)$ for =2,…,T .
3. For t=1,…,T, calculate $Y_{t}=F^{-1}\left(u_{t};n_{t},{\hat{\pi }}_{t}\right)$, where $F\left(y_{t};n_{t},{\hat{\pi }}_{t}\right)=P\left(Y\leq y_{t}|n_{t},{\hat{\pi }}_{t}\right)$ and ${\hat{\pi }}_{t}=\frac{\exp(X_{t}\beta )}{1-\exp(X_{t}\beta )}$.

The simulation is carried out with the aim of assessing the parameter estimation performance of the Copula-Based Markov chain Logistic Regression model on Binomial Time Series data. For this purpose, the data are generated from model with the parameter β and α. In this study, data are generated with logistic regression model parameter β=(−2,0.3,−0.5) and 3 Kendall-τ values, namely weak (0.2), medium (0.5) and strong (0.8). Data generation is also carried out by considering three sample sizes, n = 200, 500 and 1000. For each scenario, the simulation was repeated 500 times. Computation and simulation are implemented using R based on Algorithm 1, 2 and 3.

**Table:** 

**Algorithm 1.** Parameter estimation of copula-based Markov chain logistic regression model.

**Input**: Y,X,size **Output**: $\hat{\alpha },\hat{\beta }$ **Function**: Success Probability prob(X,β) ${\hat{\pi }}_{t}=\frac{\exp(X\hat{\beta })}{1+\exp(X\hat{\beta })}$ $$\begin{array}{c} f\left(y_{t};n_{t},{\hat{\pi }}_{t}\right)=\left(\;\;\;\;\;\begin{array}{c} n_{t}\\ y_{t} \end{array}\;\;\;\;\;\right){\hat{p}}_{t}^{y_{t}}{\left(1-{\hat{p}}_{t}\right)}^{n_{t}-y_{t}}\\ F_{t}\left(y_{t};n_{t},{\hat{\pi }}_{t}\right)=\sum_{k=0}^{y_{t}}f_{t}\left(k_{t}y_{t};n_{t},{\hat{\pi }}_{t}\right) \end{array}$$ **End Function** **Function**: Probability and cumulative distribution using binomial distribution $PDF(n,\hat{\pi })$ and $CDF(n,\hat{\pi })$ **End Function** **Function:** Marginal distribution function $u_{t}=F\left(y_{t};n_{t},{\hat{\pi }}_{t}\right),\;t=2,\ldots ,T$ $v_{t}=F\left(y_{t-1};n_{t-1},{\hat{\pi }}_{t-1}\right),\;t=1,\ldots ,T-1$ $u_{t}-1=F\left(y_{t}-1;n_{t},{\hat{\pi }}_{t}\right),\;t=2,\ldots ,T$ $v_{t}-1=F\left(y_{t-1}-1;n_{t-1},{\hat{\pi }}_{t-1}\right),\;t=1,\ldots ,T-1$ **End Function** **Function**: Log-likelihood function $LLF(Y,\;X,\;\mathrm{size},\hat{\beta ,}\hat{\alpha }\;)$ $$\begin{array}{ccc} L(\beta ,\alpha ) & = & \sum_{t=2}^{n}\text{log}\left[C\left(u_{t},v_{t};\alpha \right)-C\left(u_{t},v_{t}-1;\alpha \right)-C\left(u_{t}-1,v_{t};\alpha \right)+C\left(u_{t}-1,v_{t}-1;\alpha \right)\right]\\ & & -\sum_{t=2}^{n-1}\text{log}\left[f\left(y_{t};n_{t},{\hat{\pi }}_{t}\right)\right] \end{array}$$ **End Function Initialize**:, ${\hat{\beta }}_{0}=\left(0,0,\ldots ,0\right)$ and ${\hat{\alpha }}_{0}=2$**Function**: Parameter Estimation ParEst(Y,X,size) $$\begin{array}{c} \left(\hat{\beta },\hat{\alpha }\right)=\underset{\beta }{\text{argmax}}\;L\left(\beta ,\alpha \right) \end{array}$$ **End Function**

**Table:** 

**Algorithm 2.** Generating binomial time series data.

**Input**: β,α,T **Output**: Y,X,Size **Function**: Generate data size(T), v(T), X(T) ${\left\{\mathrm{siz}e_{t}\right\}}_{t=1}^{T}∼Unif\left(200,\;500\right).$ ${\left\{v_{t}\right\}}_{t=1}^{T}∼Unif\;\left(0,\;1\right)$ ${\left\{X1\right\}}_{t=1}^{T}∼Unif\left(70,\;100\right)$ ${\left\{X2\right\}}_{t=1}^{T}∼Unif\left(0,\;1\right)$ X(T)=(1,X1,X2) $U_{1}=v_{1}$ **For**t=2,…,T $U_{t}=C_{2|1}^{-1}\left(v_{t}\;|U=Ut-1;\alpha \right)$. ${\hat{\pi }}_{t}=\frac{\exp(X_{t}\beta )}{1-\exp(X_{t}\beta )}$ $Y_{t}=F^{-1}\left(u_{t};n_{t},{\hat{\pi }}_{t}\right)$ **End For** **End Function**

**Table:** 

**Algorithm 3.** Simulation for assessing the parameter estimation performance.

**Input**: β,α,T,B **Output**: $\bar{\beta },\bar{\alpha ,}MSE\left(\beta \right)$**,**MSE(α) **For**b=1toB Generate Y=Generate(β,α,T) Estimaste $\left(\hat{\beta },\hat{\alpha }\right)=\text{ParEst}\left(Y,\;X,\;\mathrm{size}\right)$ **End For** **Function** Mean and MSE $\left({\hat{\beta }}_{\mathrm{sim}},{\hat{\alpha }}_{sim}\right)$ $\bar{\beta }=\frac{1}{B}\sum_{i=1}^{B}{\hat{\beta }}_{i}$ $\bar{\alpha }=\frac{1}{B}\sum_{i=1}^{B}\alpha_{i}$ $MSE\left(\beta \right)=\frac{1}{B}\sum_{i=1}^{B}{\left({\hat{\beta }}_{i}-\bar{\beta }\right)}^{2}$ $MSE\left(\alpha \right)=\frac{1}{B}\sum_{i=1}^{B}{\left({\hat{\alpha }}_{i}-\bar{\alpha }\right)}^{2}$ **End Function**

Based on the previously determined copula model, namely Clayton, Gumbel and Frank, each Kendal tau correlation value is converted to the copula model parameter values. Each copula parameters for 3 Kendall-τ values are presented in Table 1.

**Table 1:** Copula model parameters for data generating.

Copula	Parameters
Clayton	α=0.5,2,8
Gumbel	α=2,6,20
Frank	α=1.25,2.5

Table 2. presents the simulation results of the copula-based Markov chain logistic regression model with Clayton copula. It can be seen that the mean of marginal parameter estimates for *T*=200,500 and 1000 are close to the actual parameter values. In addition, it can be seen that MSE(β) get smaller with increasing number of samples. For time dependence, the results of the parameter estimation with the Clayton copula model show that the mean of estimate are almost the same as the actual parameters, namely for α=0.5,2 and 8. Based on the MSE(α), it can be seen that the value tends to increase as the value of the parameter increases, but this value will also decrease with increasing sample size.

**Table 2:** Mean of estimates, MSEs (within parentheses) for clayton copula-Based Markov Chain model using logistic regression model.

T	α	${\hat{\beta }}_{0}\left(MSE\right)$	${\hat{\beta }}_{1}\left(MSE\right)$	${\hat{\beta }}_{2}\left(MSE\right)$	$\hat{\alpha }\left(MSE\right)$
200	0.5	-1.996 (0.011)	0.301 (0.000)	-5.009 (0.009)	0.521 (0.040)
	2	-2.000 (0.004)	0.300 (0.000)	-4.999 (0.003)	2.054 (0.153)
	8	-1.968 (0.003)	0.299 (0.000)	-4.987 (0.001)	6.350 (6.143)
500	0.5	-1.991 (0.001)	0.299 (0.000)	-4.981 (0.001)	0.499 (0.003)
	2	-1.994 (0.001)	0.299 (0.000)	-4.988 (0.001)	1.961 (0.060)
	8	-1.986 (0.001)	0.299 (0.000)	-4.999 (0.001)	6.653 (3.559)
1000	0.5	-1.991 (0.001)	0.299 (0.000)	-4.981 (0.001)	0.499 (0.003)
	2	-1.995 (0.000)	0.299 (0.000)	-4.989 (0.000)	1.964 (0.027)
	8	-1.989 (0.000)	0.299 (0.000)	-4.992 (0.000)	7.138 (2.086)

Table 3 presents the simulation results of the estimated parameters of the copula-based markov chain autoregressive logistic regression model with the gumbel copula model. The means of the estimated marginal parameter show that the estimations are not biased with the relatively small value of MSE(β). For estimation of the copula parameter with values α=2 and 6 it produces an average that is almost the same as the actual copula parameter. Meanwhile, for the estimation of the copula parameter with α=20 it can be seen that the mean values are smaller than the actual value with a fairly large MSE(α). However, the average value is getting closer to the true value with MSE(α) which tends to decrease as the sample *T* value increases

**Table 3:** Mean of estimates, MSEs (within parentheses) for gumbel copula-based Markov Chain model using logistic regression model.

T	α	${\hat{\beta }}_{0}\left(MSE\right)$	${\hat{\beta }}_{1}\left(MSE\right)$	${\hat{\beta }}_{2}\left(MSE\right)$	$\hat{\alpha }\left(MSE\right)$
200	2	-1.995 (0.002)	0.300 (0.000)	-4.993 (0.002)	2.013 (0.021)
	6	-2.007 (0.002)	0.301 (0.000)	-5.009 (0.002)	5,249 (1.304)
	20	-2.010 (0.001)	0.300 (0.000)	-5.004 (0.000)	11.493 (102.852)
500	2	-2.003 (0.001)	0.300 (0.000)	-5.000 (0.001)	2.000 (0.007)
	6	-2.005 (0.001)	0.300 (0.000)	-4.994 (0.000)	5,474 (0.598)
	20	-2.010 (0.002)	0.300 (0.000)	-5.005 (0.001)	11.901 (95.373)
1000	2	-1.991 (0.002)	0.299 (0.000)	-4.979 (0.002)	1.993 (0.007)
	6	-1.993 (0.000)	0.298 (0.000)	-4.975 (0.000)	5.616 (0.299)
	20	-2.008 (0.001)	0.300 (0.000)	-5.007 (0.000)	12.337 (78.051)

Similar to the copula-based markov chain autoregressive logistic regression simulation results for binomial data using the Clayton and Gumbel models, the simulation results using the copula frank show that the mean estimated marginal parameters are close to their true values, as shown in Table 4. From the table it can also be seen that the estimation of the marginal parameters shows unbiased results for n=200,500 and *1000*. For the 3 parameter values used, namely α=1.25,2 and 5, the simulation average results also approach the actual values and the MSE(α) also approaches zero as the sample size increases.

**Table 4:** Mean of estimates, MSEs (within parentheses) for Frank copula-based Markov Chain model using logistic regression model.

T	α	${\hat{\beta }}_{0}\left(MSE\right)$	${\hat{\beta }}_{1}\left(MSE\right)$	${\hat{\beta }}_{2}\left(MSE\right)$	$\hat{\alpha }\;\left(MSE\right)$
200	1.25	-2.002 (0.004)	0.300 (0.000)	-5.004 (0.004)	1.225 (0.203)
	2	-2.001 (0.004)	0.300 (0.000)	-4.998 (0.004)	1.972 (0.203)
	5	-2.001 (0.002)	0.300 (0.000)	-5,003 (0.002)	4.951 (0.283)
500	1.25	-2.000 (0.002)	0.300 (0.000)	-5.001 (0.002)	1.228 (0.074)
	2	-2.002 (0.002)	0.300 (0.000)	-5.000 (0.002)	1.987 (0.076)
	5	-2.002 (0.001)	0.300 (0.000)	-5,002 (0.001)	4.959 (0.107)
1000	1.25	-2.001 (0.001)	0.300 (0.000)	-4.999 (0.001)	1.237 (0.039)
	2	-2.002 0.001))	0.300 (0.000)	-4.999 (0.000)	1.984 (0.038)
	5	-2.000 (0.001)	0.300 (0.000)	-4.999 (0.000)	5.013 (0.054)

From the simulation results of the copula-based Markov chain autoregressive logistic regression model for binomial data with copula Clayton, Gumbel and Frank, it can be concluded that parameter estimation using the MLE method on the copula-based Markov chain autoregressive logistic regression model for binomial data provides unbiased estimates for β and α. This can be seen from the mean estimated parameter value which are close to the actual value and the MSE values which are close to zero when the sample size are getting bigger.

### Apllication of copula-based logistic autoregressive regression model

The Copula-Based Logistic Autoregressive Regression Model is implemented in Program R and applied to the human influenza data set in Singapore obtained from [22]. Singapore is a tropical country and the weather is considered to have an important role in the transmission of influenza. It has been shown previously that the global dynamics of influenza outbreaks is determined by seasonal fluctuations in climatic factors such as temperature, amount of rainfall and relative humidity. The dependent data are the number of positive influenza samples and the number of monthly influenza surveillance specimens for the period from October 2011 to March 2014. The independent data are the monthly temperature (in degrees Celsius), the amount of precipitation (in mm/month), and vapor pressure (in hPa).

**Fig. 1:**
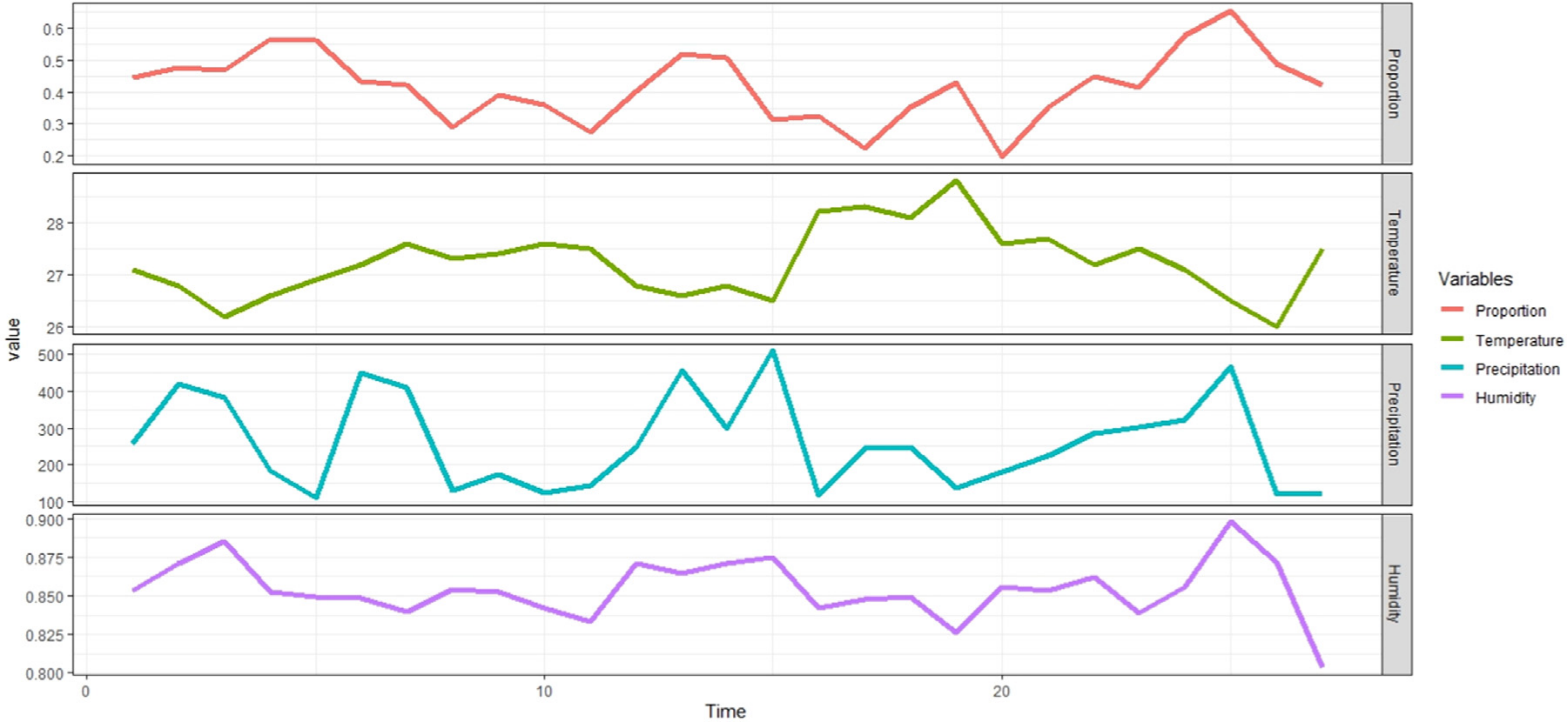
Time series plot of monthly propotion of influenza surveillance specimens humidity, precipitation and temperature in Singapore.

Fig. 1 shows time series plots of the dependent and independent variables from a case study of influenza data in Singapore. From the figure it can be seen that the proportion of people affected by confirmed influenza to the specimens examined was in the range of 0.20 %–0.65 % with the lowest in July 2013 and the largest percentage in December 2013. The figure above also shows that the proportion reached its highest level in December or January is also characterized by a decrease in air temperature and an increase in rainfall and humidity. Therefore, it can be suspected that cases of influenza have a negative relationship with temperature and have a positive relationship with rainfall and air humidity.

### Results and analyses

In this study it is assumed that the data on people affected by confirmed influenza on the specimens examined had a binomial distribution, so that the marginal distribution used was binomial with a logistic regression model. For the time dependence structure, the Markov model used is the Clayton, Gumbel and Frank bivariate function. The parameter estimation results of each model are presented in Table 5.

**Table 5:** Parameter estimates for copula-based Markov Chain logistic autoregressive regression models fit to the human influenza data.

Model	${\hat{\beta }}_{0}$	${\hat{\beta }}_{1}$	${\hat{\beta }}_{2}$	${\hat{\beta }}_{3}$	$\hat{\alpha }$	LL	MSE	MAPE
Clayton	-0.00053	-0.01588	0.00074	-0.00043	0.14928	-154.2926	17.5769	0.0495
Gumbel	-0.00054	-0.01620	0.00213	-0.00043	2.67498	- 238.2980	182.1538	0.2168
Frank	-0.00047	-0.01528	0.00073	-0.00036	2.19288	**- 153.0779**	**5.5769**	**0.0468**

Table 5 shows a comparison of the BLR model, and the copula-based Markov chain logistic regression model with Clayton, Gumbel and Frank Copula. The results show that the probability parameter estimations $\hat{\beta }$ for three copulas are nearly the same with time dependencies are 0.14928, 2.67498 and 2.19288 for Clayton, Gumbel and Frank Copula respectively. The higest log-likelihood value is produced by Frank Copula which is also followed by the smallest MSE and MAPE. Therefore, based on the log-likelihood, MSE and MAPE values, the best copula to model the dependency structure on human influenza data is the Frank copula.

After determining the best copula for the copula-based Markov chain logistic regression model, the next step is to interpret the parameter estimations. Before discussing the parameters of the copula model in the time dependent structure, the parameters of the marginal model are interpreted first. The copula-based Markov Chain Logistic Autoregressive Regression model with Frank Copula shows the negative impact of increasing air temperature due to ${\hat{\beta }}_{1}<0$. In addition to temperature, the coefficient of humidity is also negative which results in decreased cases of influenza with increasing humidity. For the precipitation variable, the parameter coefficient is positive with ${\hat{\beta }}_{2}=0.0007$ which can be interpreted that the more rainfall, the chance of an increase in influenza cases also increases. However, in this model, ${\hat{\beta }}_{3}$ is negative, indicating that increasing relative humidity reduces the probability of adding influenza cases.

The parameter values are less than one and relatively small, it is expected that the independent variables has no significant effect on the dependent variable. Because our focus in this study is to model and estimate parameters, the significance test cannot be carried out. However, because our focuses in this research are building models and parameter estimates, these parameters are still included in the model. Therefore, the marginal distribution used in determining the time dependence on influenza data has the success proportion given as:

(33) $$\pi =\frac{e^{-0.00047\;-\;0.01528Temp+0.00073Precip\;-0.00036Humid}}{1+e^{-0.00047\;-\;0.01528Temp+0.00073Precip\;-0.00036Humid}}$$

Furthermore, the interpretation of the dependency structure is in the form of autocorrelation. The best model for influenza data is copula-based Markov chain logistic regression model with Frank Copula function. Consequently, it can be concluded that the influenza data has a symmetrical time dependence and has no dependence on the lower and upper tails. In Table 4, the copula parameter value for the Frank copula model is 2.19288 and when expressed in the Kendals-tau value is 0.2320. It can be stated that today's influenza activity has a weak effect on tomorrow's influenza cases. Therefore, the conditional expectation $Y_{t}$ given $Y_{t-1}$ in the copula-based Markov chain logistic regression model can be expressed in the following equation:

(34) $$\mathrm{Pr}\left(Y_{t}=y_{t}|Y_{t-1}=y_{t-1}\right)=e^{-2.19288v{\left[\left(e^{-2.19288}-1\right){\left(e^{\theta 2.19288}-1\right)}^{-1}+\left(e^{2.19288v}-1\right)\right]}^{-1}}$$

where v is the binomial cumulative distribution with the probability of success in Eq. (33).

**Fig. 2:**
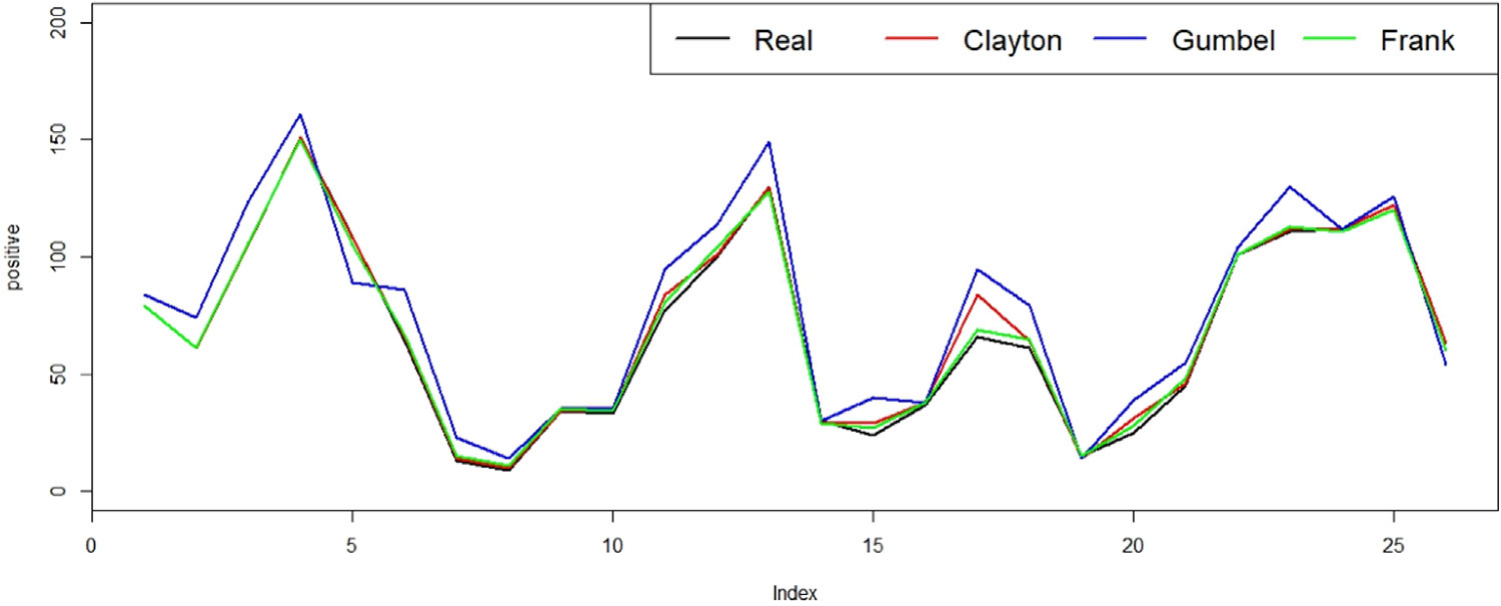
Predicted values using copula-based Markov chain Logistic regression models to human influenza Data in Singapore.

Fig. 2 displays the predicted values of the Markov chain logistic regression model with Clayton, Gumbel and Frank Copula for the number of positive influenza in Singapore based on climatic factors. The real data is marked with black lines, while the prediction results using Clayton, Gumbel and Frank copulas are marked with red, blue and green lines respectively. Fitted values with the copula model based on Markov chain show results that are almost similar to the actual values. However, the Markov chain model with frank copulas works better than the Markov model with Clayton and Gumbel copulas.

Furthermore, the copula-based Markov chain model are compared with other binomial data models, namely the Binomial Logistic Regression (BLR) model for ordinary binomial response data which does not assume autocorrelation and GLMM for binomial time series models which assumes autocorrelation expressed by latent processes. The following are the results of parameter estimation from the BLR, GLMM and copula-based Markov Chain models using Frank Copula:

Table 6 shows the log-likelihood, MSE and MAPE values for the BLR, GLMM and Copula-Based models. The results demonstrate that GLMM has the largest log-likelihood as well as the highest MSE and MAPE. The log-likelihood, MSE, and MAPE values from the BLR model are smaller than those from the GLMM. While the copula-based model has the smallest log-likelihood, it also has a substantially lesser MSE and MAPE than the other two models. Consequently, GLMM is the best model based on the log-likelihood value, whereas the Copula-based Markov chain model is the best model based on the errors. This is perhaps explained by the fact that integrating temporal dependency in a copula-based Markov chain reduces modelling mistakes. In other words, if we require a model with accurate prediction values, the copula-based model with Frank Copula is the best option.

**Table 6:** The result comparison of BLR, GLMM and copula-based Markov Chain model.

Model	LL	MSE	MAPE
BLR	-130.4072	170.2692	0.1864
GLMM	**-101.2764**	237.0713	0.2353
Copula-Based	- 153.0779	**5.5769**	**0.0468**

**Fig. 3:**
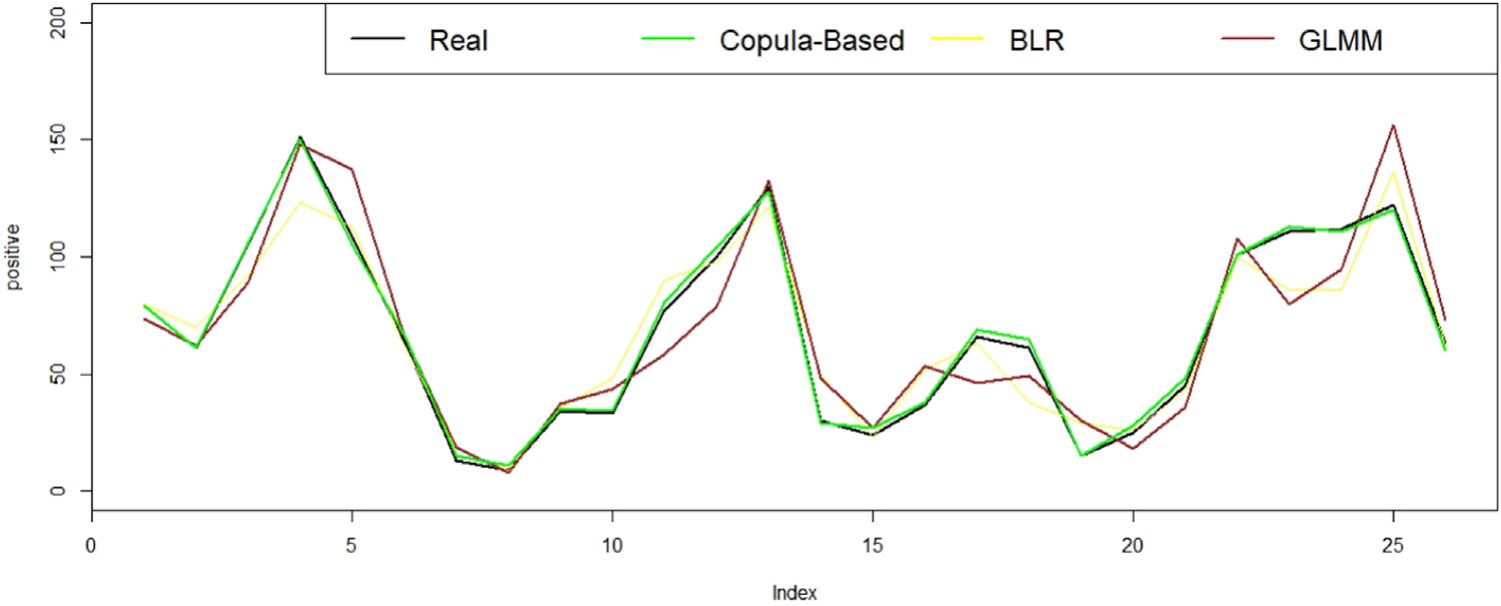
Predicted values using the copula-based Markov Chain, BLR and GLMM for binomial time series data of human influenzain Singapore.

Fitted values further shown in Fig. 3, where the predicted value of the copula-based Markov chain is nearly identical to the actual value. This appears to be connected to the assumption that considering temporal dependency in a Copula-based Markov Chain can reduce modelling errors. This is in accordance with the statement of Alqawba and Diawara (2020) that ignoring time dependencies can give inaccurate results [9]. Therefore, in terms of MSE and MAPE values, our proposed approach can improve prediction accuracy and provide a better model for influenza data than the BLR model and GLMM.

## Conclusions

Copula-based Markov chain model using logistic regression models can be used in modelling binomial time series data with covariate variables. This model also fulfils the asymptotic properties of unbiased and normality. Based on the simulation results, it is concluded that the MLE method provides an unbiased estimate of the copula-based autoregressive logistic regression model. The copula-based Markov chain model can also be applied to data on the influence of weather on influenza cases in Singapore and it can be concluded that the best copula model is the Frank Copula which has a symmetrical time dependence and has no dependence on the lower and upper tails.

Based on theoretical development, simulation and application, the copula-based Markov chain logistic regression model has several advantages, including not only being able to see the relationship between independent and dependent variables but also providing estimates of the inter-time dependence of the dependent variable; estimating parameters directly using the MLE method; time dependencies in the copula model can increase the accuracy of the prediction model compared to the latent process binomial time series model. This research only proposes parameter estimation in the copula-based Markov chain logistic regression model. Further research is needed to develop the use of models with additional statistical inferences, such as confidence intervals and hypothesis testing.

## CRediT authorship contribution statement

**Pepi Novianti:** Conceptualization, Methodology, Data curation, Software, Visualization, Writing – original draft. **:** Conceptualization, Methodology, Validation, Writing – review & editing, Supervision. **Dedi Rosadi:** Conceptualization, Methodology, Validation, Software, Writing – review & editing, Supervision.

## Declaration of Competing Interest

The authors declare that they have no known competing financial interests or personal relationships that could have appeared to influence the work reported in this paper.

## Data Availability

I used data from sources that I explain in my manuscript.
